# Effects of *Satureja montana* L. and *Origanum vulgare* L. Hydrolates in Rabbit Burn Wound Model: Evaluation of Inflammatory, Antioxidant Activity, and Pro-Regenerative Properties in the Skin

**DOI:** 10.3390/ijms26178628

**Published:** 2025-09-04

**Authors:** Grigory Demyashkin, Alibek Tokov, Dmitriy Belokopytov, Vladimir Shchekin, Tatyana Borovaya, Daniel Lukash, Daniil Yuferov, Nina Kulchenko, Vadim Tarasov, Ekaterina Blinova, Dibakhan Tsomartova, Peter Shegai, Andrey Kaprin

**Affiliations:** 1 Department of Digital Oncomorphology, National Medical Research Centre of Radiology, 2nd Botkinsky pass, 3, Moscow 125284, Russia; beldimbur@gmail.com (D.B.); dr.shchekin@mail.ru (V.S.); tbor27@yandex.ru (T.B.); dr.shegai@mail.ru (P.S.); kaprin@mail.ru (A.K.); 2 Laboratory of Histology and Immunohistochemistry, Institute of Translational Medicine and Biotechnology, I.M. Sechenov First Moscow State Medical University (Sechenov University), Trubetskaya, 8/2, Moscow 119048, Russia; daniellukas-golitsin@yandex.ru (D.L.); yuferov.dy@dvfu.ru (D.Y.); tarasov_v_v_2@staff.sechenov.ru (V.T.); blinova_e_v@staff.sechenov.ru (E.B.); tsomartova_d_a@staff.sechenov.ru (D.T.); 3 Research and Educational Resource Center for Immunophenotyping, Digital Spatial Profiling and Ultrastructural Analysis Innovative Technologies, Peoples’ Friendship University of Russia (RUDN University), Miklukho-Maklaya, 6, Moscow 117198, Russia; tokov.alibek@bk.ru (A.T.); kle-kni@mail.ru (N.K.); 4 Department of Fundamental Medicine, Institute for Physics and Engineering in Biomedicine, National Research Nuclear University MEPhI, Kashirskoe shosse, 31, Moscow 115409, Russia; 5 Department of Urology and Operative Nephrology, Peoples’ Friendship University of Russia (RUDN University), Miklukho-Maklaya, 6, Moscow 117198, Russia

**Keywords:** burns, *Satureja montana* L., *Origanum vulgare* L., plant extracts, inflammation, regeneration, redox status

## Abstract

Burn injuries are among the most difficult skin lesions to manage, as they trigger intense inflammatory responses and oxidative stress, which often impair angiogenesis, delay epithelialization, and increase the risk of chronic non-healing wounds. Hydrolates of *Satureja montana* L. and *Origanum vulgare* L., rich in antioxidant and anti-inflammatory compounds, offer a promising natural alternative for wound management. This study investigated their effects on local redox and inflammatory status in full-thickness burn wounds. Male rabbits (n = 5 per group) received full-thickness burns and were assigned to control, untreated, conventional treatment (Levomekol liniment, boric acid, and Betadine-soaked gauze dressings), *Satureja montana* L. hydrolate, and *Origanum vulgare* L. hydrolate groups. Skin samples were collected on days 3, 7, and 14. ELISA was used to quantify redox (MDA, SOD, GSH) and inflammation (TNF-α, IL-1, IL-10) markers. Histochemical (H and E, Masson’s trichrome) and immunohistochemical (CD-45) analyses, plus the Greenhalgh score, were used to assess wound healing. Burn injuries significantly altered the redox status in all treated and untreated groups. The hydrolates reduced MDA and restored SOD/GSH levels, with *Satureja montana* L. showing the most pronounced effects. *Satureja montana* L. hydrolate modulated pro- and counter-inflammatory cytokines (decreasing IL-1/TNF-α, upregulating IL-10). An assessment of local cellular immunity showed the most prominent decrease in CD45+ cell counts in groups treated with *Satureja montana* L. and *Origanum vulgare* L. hydrolates. This study provides promising evidence that *Satureja montana* L. and *Origanum vulgare* L. hydrolates offer promise as topical therapies for burn wounds by modulating ROS production and local inflammatory status and by improving wound healing, with *Satureja montana* L. hydrolate exhibiting the most pronounced therapeutic effect.

## 1. Introduction

Burn wounds are among the most challenging skin lesions to treat, requiring prompt and effective intervention to minimize complications, including local inflammation and delayed healing. Despite the clinical relevance of this issue, available therapeutic options remain limited and are focused mainly on restoring fluid and electrolyte balance, providing pain relief, immunization, and antibiotic prophylaxis [1]. These interventions, however, are largely supportive and do not directly promote tissue regeneration. Moreover, the growing prevalence of multidrug-resistant pathogens, such as methicillin-resistant *Staphylococcus aureus* (MRSA), vancomycin-resistant *Enterococci* (VRE), and carbapenem-resistant *Enterobacteriaceae* (CRE), has further reduced the efficacy of conventional antibiotic therapies [2,3]. As a result, medicinal plant-based preparations have gained increasing attention [4], with several studies demonstrating their therapeutic potential as effective alternatives or adjuncts to classical treatment approaches in cases of moderate burns.

Among the plant-based preparations used in the treatment of non-burn wounds, phytopreparations based on *Satureja montana* L. and *Origanum vulgare* L. have shown effectiveness due to the presence of carvacrol, gammaterpinene, and p-cymene, which possess a range of beneficial properties. While these compounds are known for their antibacterial, immunomodulatory, and pro-regenerative effects [5,6,7], this study specifically focuses on their antioxidant and anti-inflammatory properties in the context of burn wound healing. The antimicrobial properties of these compounds are particularly relevant in the context of rising antibiotic resistance, offering a potential alternative or adjunct to conventional antibiotics [8]. Recent studies have demonstrated that essential oils and hydrolates from these plants exhibit significant antimicrobial activity against both Gram-positive and Gram-negative bacteria, including antibiotic-resistant strains [9].

Despite the fact that ROS generation is one of the most important mechanisms of defense against pathogens [10], the excess of reactive oxygen species resulting from thermal damage adversely affects both the proliferation of epidermal cells and local immune status, paralyzing neutrophil activity. There is evidence to suggest that suppressing excessive ROS production helps to protect against subsequent wound infectious complications [11].

For the phytotherapeutic treatment of burn wounds, hydrolates—water-based extracts obtained by steam distillation—are among the most rational medicinal forms for application [3].

We hypothesize that hydrolate phytopreparations based on *Satureja montana* L. and *Origanum vulgare* L. are able to accelerate the healing of burn wounds, including by modulating local production of reactive oxygen species towards pro-regenerative and anti-pathogenic properties.

### Research Objective

Aim of the study: This study aimed to assess the effects of *Satureja montana* L. and *Origanum vulgare* L. hydrolate treatment on the local redox and inflammatory system status of skin following grade III burn injury induced with a pre-heated copper plate. The objective was to elucidate therapy-dependent effects on the skin’s oxidative and inflammatory status.

## 2. Results

### 2.1. Chemical Composition of Hydrolates

The chemical composition of the *Satureja montana* L. and *Origanum vulgare* L. hydrolates was determined by GC–MS analysis, revealing distinct yet characteristic volatile profiles for each. As shown in Table 1 and Table 2, the extracts demonstrated a diverse phytochemical composition, supporting their overall quality.

For the *Satureja montana* L. hydrolate (Table 1), carvacrol emerged as the dominant compound, constituting 89.2% of the total volatile fraction. Other significant components, although in much lower concentrations, included p-cymene (3.1%), thymol (2.8%), and γ-terpinene (1.6%), alongside minor contributions from linalool, borneol, terpinen-4-ol, 1-octen-3-ol, myrcene, α-terpineol, and 1,8-cineole. The presence of these compounds, even in smaller amounts, contributes to the overall synergistic effects and therapeutic potential of the *Satureja montana* hydrolate.

In contrast, the *Origanum vulgare* L. hydrolate (Table 2) also featured carvacrol as the most abundant compound, albeit at a lower concentration of 43.7%, compared to *Satureja montana.* Notably, a substantial amount of thymol was present (18.6%), indicating a more balanced phenolic profile between these two key monoterpenes. Other prominent constituents included p-cymene (9.4%) and γ-terpinene (7.8%), followed by sabinene, linalool, β-myrcene, α-pinene, terpinen-4-ol, α-terpinolene, and 1,8-cineole.

### 2.2. Assessment of Inflammatory and Oxidative Stress Markers

**Figure 1:**
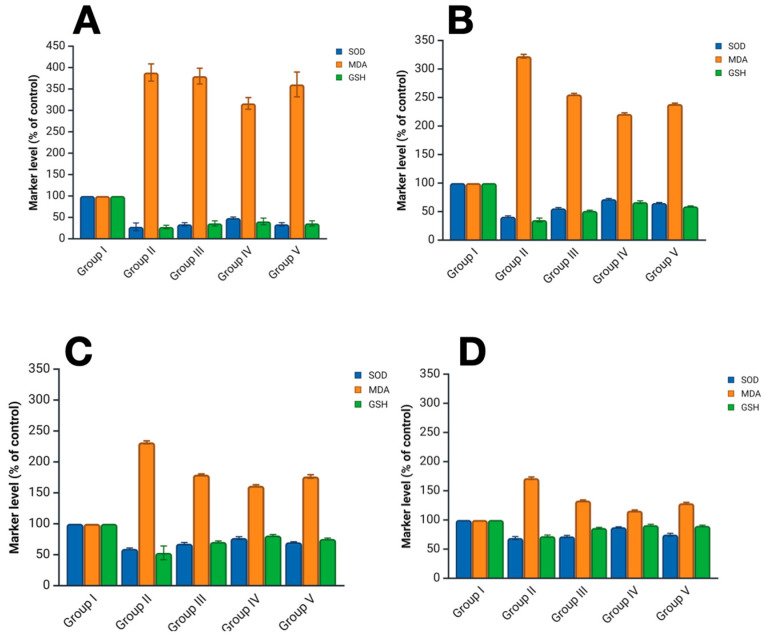
Redox status marker levels: (**A**) baseline; (**B**) on day 3; (**C**) on day 7; and (**D**) day 14. Data are presented as % of control values. SOD—superoxide dismutase; MDA—malondialdehyde; GSH—glutathione. Group labeling corresponds to the study design: Group I—intact control; Group II—untreated burn model; Group III—conventional treatment; Group IV—*Satureja montana* L. hydrolate; Group V—*Origanum vulgare* L. hydrolate.

During the assessment of the oxidative status in homogenized skin samples in the burn wound area using enzyme-linked immunosorbent assay, significant changes in key redox system markers were demonstrated in all experimental groups compared to the control group. Based on the quantitative ELISA data on the levels of SOD, MDA, and GSH, median values were calculated for each of the markers in all groups, after which, for convenience, the indicators of the control group on days 3, 7, and 14 were taken as 100% (Figure 1).

Throughout the experiment, a significant increase in the level of malondialdehyde (MDA), a marker of lipid peroxidation, was found in skin homogenates with a burn wound (groups II–V). In contrast, the levels of superoxide dismutase (SOD) and glutathione (GSH) in these groups decreased markedly.

On day 3, the highest achieved level of MDA was observed in group II—a 3.2-fold increase compared to the control group (*p* < 0.05). In group III, which received conventional treatment, there was a 2.5-fold elevation of MDA. A lower MDA value was found in skin homogenates of the burn wound treated with hydrolates of *Satureja montana* L. and *Origanum vulgare* L., which amounted to 2.21-fold and 2.39-fold compared to the control in groups IV and V (*p* < 0.05).

Subsequently, a decrease in the level of MDA was noted in all groups on days 7 and 14. However, the most rapid restoration of MDA levels was found in group IV (especially) and group V.

Analysis of skin homogenates from the burn wound revealed a decrease in SOD and GSH levels on day 3 compared to control values.

At this point, the worst SOD value was in group II (burn wound without treatment), with 41% of control values (*p* < 0.05).

Even on day 3, the best SOD values—the most powerful protective antioxidant enzyme—were found in skin homogenates of the burn wound treated with hydrolates of *Satureja montana* L. and *Origanum vulgare* L. Moreover, with the use of *Satureja montana* L. hydrolates, SOD levels demonstrated values closest to the control.

Throughout the experiment, SOD levels showed themselves ambiguously and depended on the condition of the burn wound and the treatment performed. The rate of increase in the SOD indicator in skin homogenates of the burn wound treated with hydrolates, especially *Satureja montana* L., exceeded that in the group with conventional therapy (Group 3); by day 14, the SOD level was 88% (*p* < 0.05) relative to control values. Groups III and V demonstrated comparable results—72% and 75% for SOD, and 86% and 90% for GSH relative to the control (*p* < 0.05).

On day 3, the lowest level of GSH was observed in group II—a threefold decrease compared to that of the control group (*p* < 0.05). In group III, which received conventional treatment, the GSH level decreased to 52%. A higher GSH value was found in skin homogenates of the burn wound treated with hydrolates of *Satureja montana* L. and *Origanum vulgare* L.; they amounted to 66% and 59% compared to the control in groups IV and V (*p* < 0.05).

Subsequently, an increase in GSH levels was noted in all groups on days 7 and 14. However, the most rapid restoration of GSH levels was found in group IV and group V.

According to the levels of MDA, SOD, and GSH obtained using ELISA, our results indicate that conventional therapy and phytotherapy with hydrolates based on *Satureja montana* L. and *Origanum vulgare* L. significantly reduced the level of reactive oxygen species in the burn wound, with the best achieved indicators in skin homogenates of the burn wound treated with *Satureja montana* L. hydrolate (group IV).

**Figure 2:**
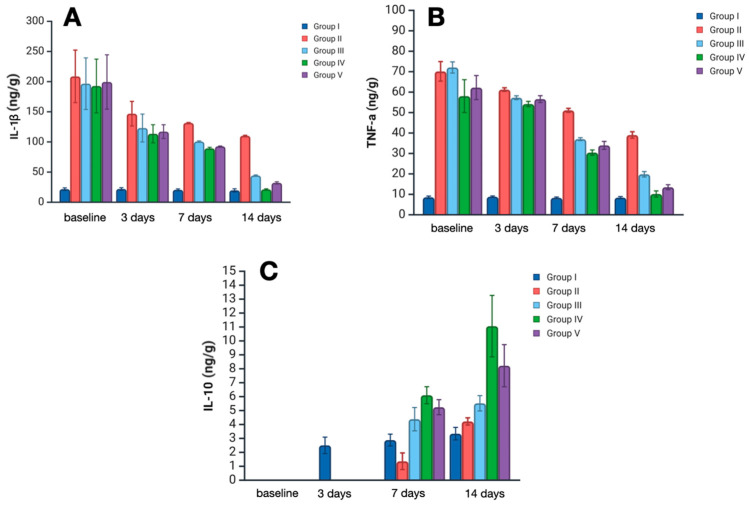
Inflammatory marker levels at all experiment time-points: (**A**) IL-1β—interleukin-1β; (**B**) TNF-α—tumor necrosis factor alpha; and (**C**) IL-10—interleukin-10. Group labeling corresponds to the study design: Group I—intact control; Group II—untreated burn model; Group III—conventional treatment; Group IV—*Satureja montana* L. hydrolate; Group V—*Origanum vulgare* L. hydrolate.

Quantification and analysis of inflammatory markers in skin sample homogenates revealed a significant impact of the treatment strategy on levels of interleukin 1 (IL-1), tumor necrosis factor alpha (TNF-α)—the main pro-inflammatory cytokines, and on the level of interleukin 10 (IL-10)—a counter-inflammatory cytokine (Figure 2).

IL-1 is one of the key mediators of pro-inflammatory states at local and systemic levels, secreted mainly by immune cells, or any another cell dying with pyroptosis. On day 3, Il-1 levels increased significantly in all experimental groups, representing acute inflammation. However, there were remarkably lower levels of IL-1 in treatment groups III–V compared with group II (no treatment) (*p* < 0.01). The considerable dispersion of values at this time point limits the ability to establish statistically significant differences among the different treatments; however, the lowest median level of IL-1 was observed in group IV—119.2 ng/g (*Satureja montana* L. hydrolate treatment). On day 7, levels of IL-1 decreased in all experimental groups, with the most prominent lowering in groups IV and V—89 ng/g and 92.5 ng/g, respectively (*p* < 0.05). On day 14, the median IL-1 level in group IV was almost equal to that of the control group—21 ng/g, which probably reflects the almost complete abatement of the inflammatory process. A less pronounced reduction was observed in groups 3 and 4. In group 2, IL-1 levels remained significantly elevated on day 14, exceeding the control values by more than fivefold.

TNF-α is a comprehensive pro-inflammatory marker that orchestrates all stages of the acute and chronic inflammatory process. Its levels on day 3 were greatly elevated in the skin samples of groups II–V, representing active inflammation after burn injury. However, on day 7, TNF levels significantly decreased in all treatment groups when compared with group II (*p* < 0.01). The lowest median level was observed in samples of group IV and V—30.9 and 33.3 ng/g, respectively. On day 14, TNF levels in groups IV and V were close to normal; moreover, a lower level was observed in group IV.

Il-10 is a counter-inflammatory cytokine, notably suppressing the secretion of IL-1 and TNF synthesis and secretion. On day 3, IL-10 levels in groups II-V were below detection limits and therefore could not be assessed correctly. On day 7, IL-10 was upregulated, with the most prominent elevation in group IV (*Satureja montana* L. treatment) at 6.1 ng/g. On day 14, the median level of IL-10 in group IV was more than 3 times higher than that in the control group—11 ng/g against 3.3 ng/g. In groups III and V, IL-10 levels were significantly lower than in group IV, with 5.6 ng/g in the classic treatment group and 8.3 ng/g in the *Origanum vulgare* L. hydrolate treatment group (*p* < 0.05). Although the IL-10 levels in group II were near the control, the simultaneous presence of IL-1 and TNF-α implies an active inflammatory state.

### 2.3. Morphological Block

Macroscopically, in all excised skin samples from groups II–V, the thermal burn area was reddish-brown, dry, and lacked elasticity. On day 7, groups II and III exhibited a small amount of greenish-yellow exudate on the surface of the burn wound, which persisted partially until the end of the experiment. In contrast, animals in groups IV (especially) and V showed macroscopic signs of burn wound healing starting as early as day 7, with complete replacement of the wound defect by day 14; signs of infection were absent. The slowest burn wound healing rate was observed in animals of group II. This observation was further supported by visual assessment of wound closure and re-epithelialization from images taken throughout the experimental period.

**Figure 3:**
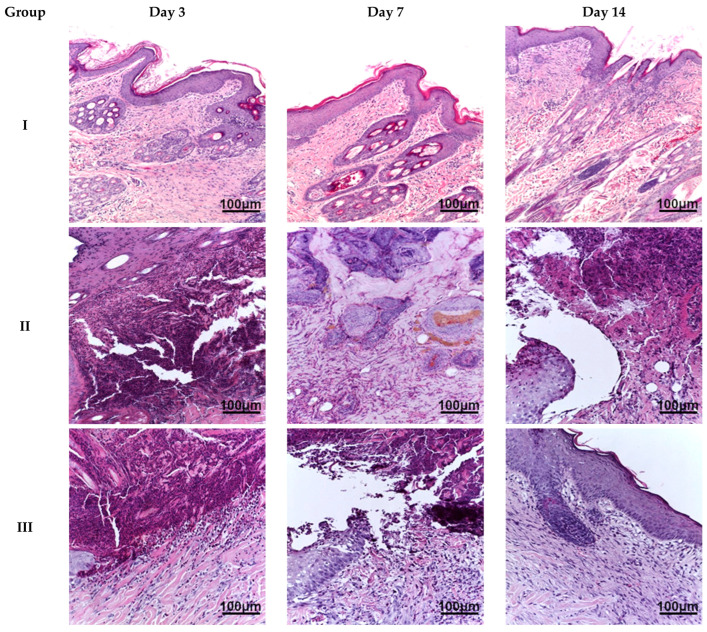

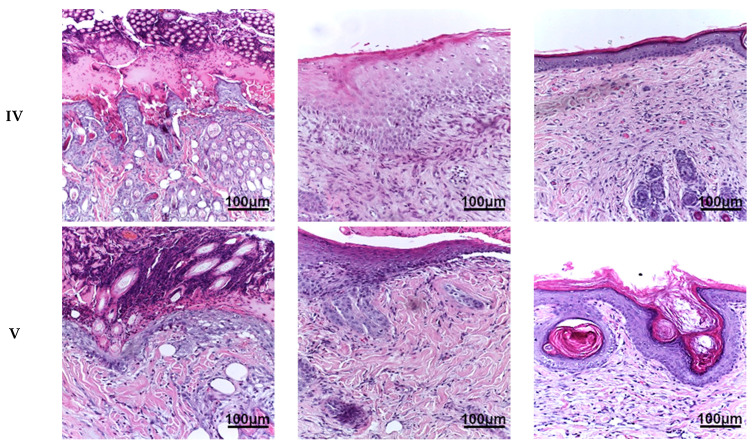
Skin of animals on days 3, 7, and 14. Staining: hematoxylin and eosin, magnification ×200. Group labeling corresponds to the study design: Group I—intact control; Group II—untreated burn model; Group III—conventional treatment; Group IV—*Satureja montana* L. hydrolate; Group V—*Origanum vulgare* L. hydrolate.

Microscopic examination of the burn wound defect in all animals on day 3 revealed a large amount of necrotic tissue debris, diffuse, extensive inflammatory infiltration (predominantly by cells of the granulocytic-monocytic lineage), isolated areas of granulation tissue formation, and hyperemia of blood vessels. In the region of the wound edges, reactive changes in the shape of epithelial cell nuclei and isolated apoptotic bodies were observed. On day 7, animals in all groups exhibited a morphological appearance of burn wound healing with signs of regeneration and tissue repair. However, animals in group IV showed the most pronounced regenerative changes in the wound defect, with re-epithelialization and reduced inflammatory infiltration. Less prominent changes were observed in the biopsy samples from animals in groups III and V. The least pronounced signs of regeneration and tissue reorganization were found in the biopsy material from animals in group II. On day 14, burn wounds in the animals in group IV were almost completely regenerated, exhibiting subtotal wound re-epithelialization and minimal or no inflammatory dermal infiltration. Similar changes were observed in group V, although focal lymphoid infiltration was present in the sub-epidermal layers. In group III, we observed moderate wound healing with persistent active inflammation. In group II, only the beginning stages of wound re-epithelialization were evident (Figure 3).

**Figure 4:**
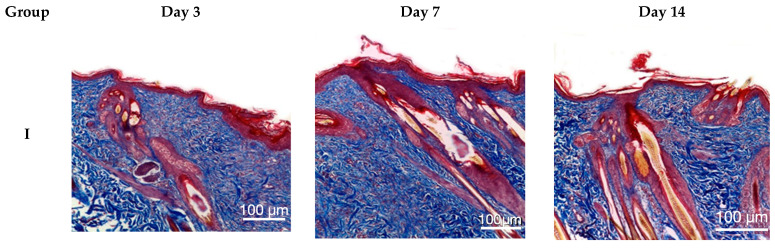

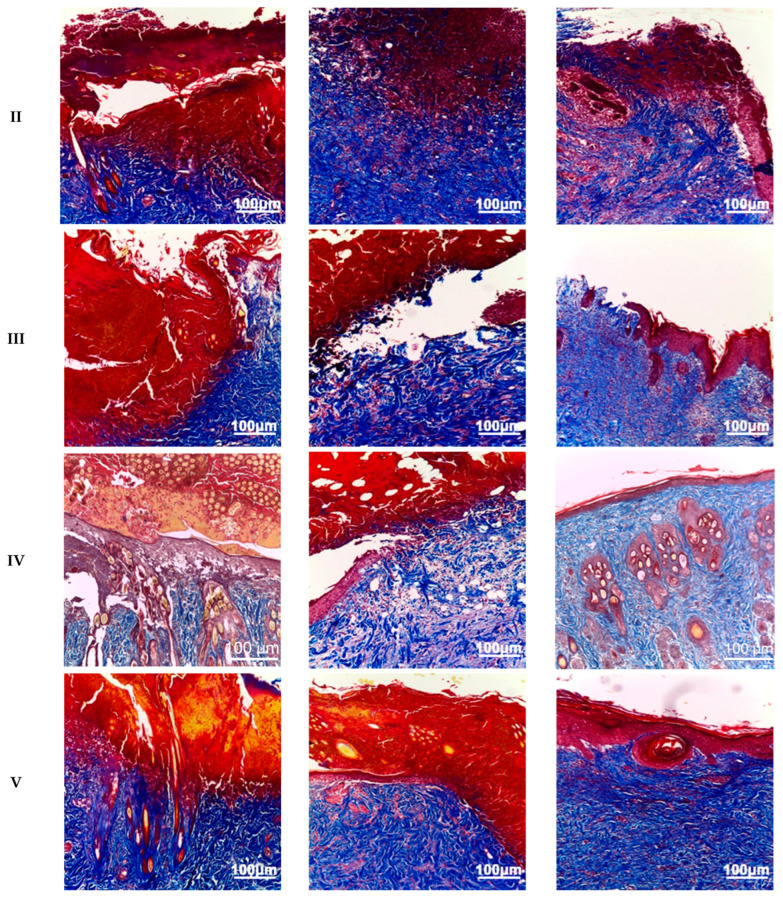
Skin of experimental animals on days 3, 7, and 14. Staining: Masson’s trichrome; magnification ×200. Group labeling corresponds to the study design: Group I—intact control; Group II—untreated burn model; Group III—conventional treatment; Group IV—*Satureja montana* L. hydrolate; Group V—*Origanum vulgare* L. hydrolate.

Following Masson’s trichrome staining, animals in groups II–V exhibited virtually complete disorganization of collagen and elastin fibers within the burn wound on day 3. By day 7, animals in groups II–V showed reorganization of the extracellular matrix, characterized by areas of collagen fiber formation and a reduction in the amount of denatured collagen. Group IV animals displayed the greatest collagen content in the burn wound area, with numerous small zones of collagen fibers, primarily located at the periphery. On day 14, the reticular dermis in group IV animals was almost completely restored, while animals in groups III and V exhibited less pronounced remodeling. Group II animals demonstrated the least synthesis of collagen fibers by day 14, with only sparse, small, and isolated collagen fibers present (Figure 4).

### 2.4. Immunohistochemical Staining

**Figure 5:**
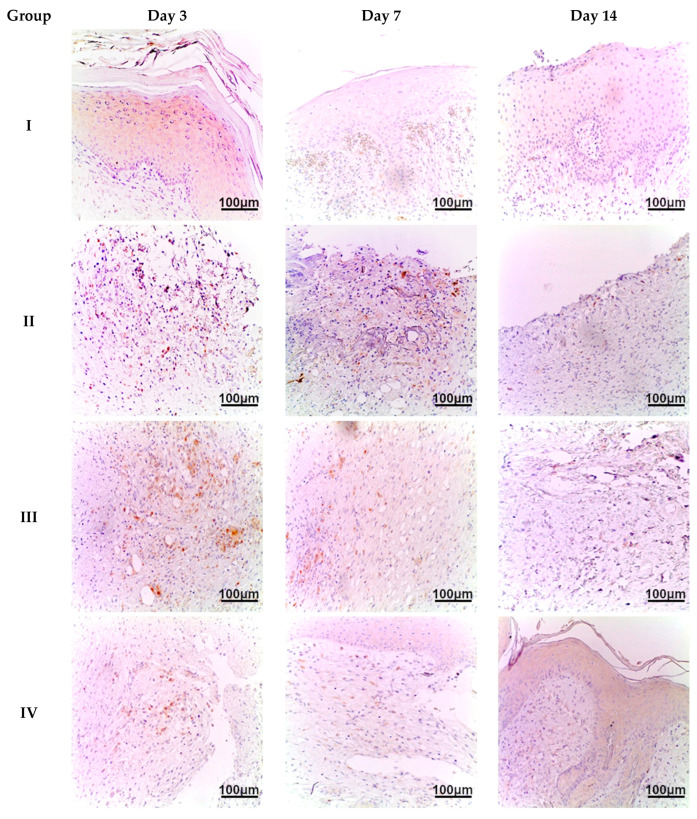

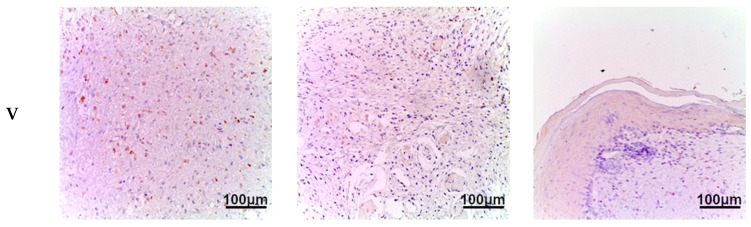
Skin of experimental animals on days 3, 7, and 14. Immunohistochemical staining with antibodies to CD45; magnification ×400. Group labeling corresponds to the study design: Group I—intact control; Group II—untreated burn model; Group III—conventional treatment; Group IV—*Satureja montana* L. hydrolate; Group V—*Origanum vulgare* L. hydrolate.

CD45 is a common leukocyte marker used for the identification of immune cells such as neutrophils, monocytes, macrophages, lymphocytes, etc. Therefore, it can be used as an integrated marker of the cellular component of inflammatory response. At the first time point (day 3), we observed an extensive inflammatory infiltration at the burn injury site, indicating acute inflammation due to significant thermal tissue destruction in all experimental groups (II–V). The density of CD45+ cells per mm^2^ was highest in group II (no treatment)—617/mm^2^. On day 7, the number of CD45+ cells decreased, especially in samples of group IV and V—72/mm^2^ and 129/mm^2^, respectively. A less prominent decrease was observed in the skin samples of group II—about 9% lower compared to the day 3 results—562/mm^2^. On day 14, immune cell density was lowest in the group IV samples—17/mm^2^ (*Satureja montana* L. treatment). Group V and III samples (*Origanum vulgare* L. and classic treatment, respectively) had higher CD45+ cell density—28/mm^2^ and 37/mm^2^, although this was still much lower than that seen in group II (no treatment). The analysis of the distribution of CD45+ cells in the skin indicates a decrease in immune cells over time, especially in group IV (*Satureja montana* L. hydrolates) compared to that of other groups (Figure 5).

### 2.5. Integrated Burn Wound Regeneration Assessment

**Figure 6:**
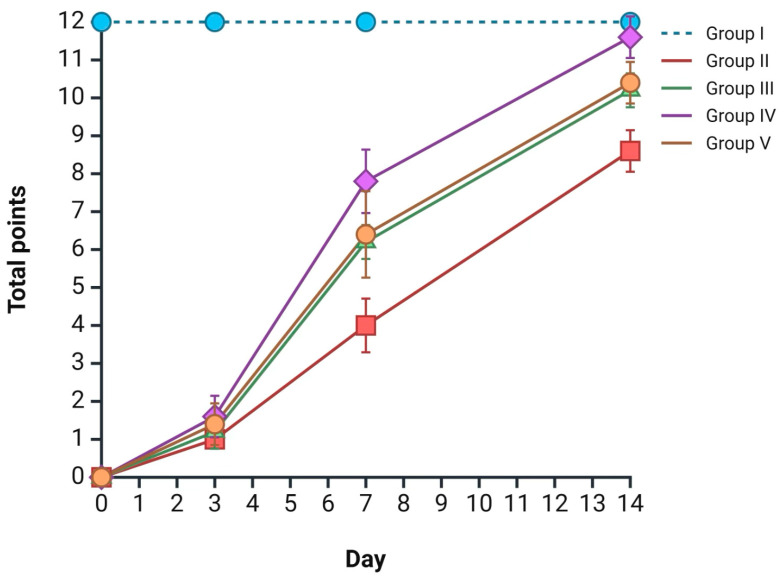
Integrated wound healing assessment at all time-points using the Greenhalgh score. The maximum score of 12 is derived from the summation of individual parameter scores, each ranging from 0 to 4, as detailed in the Materials and Methods section. Group labeling corresponds to the study design: Group I—intact control; Group II—untreated burn model; Group III—conventional treatment; Group IV—*Satureja montana* L. hydrolate; Group V—*Origanum vulgare* L. hydrolate.

According to the aforementioned morphological data, an integrated assessment of burn wound healing rates was performed for each group at the designated time points using the Greenhalgh scale; all data are presented in Figure 6 The most intensive wound regeneration process was observed in group IV (*Satureja montana* L. hydrolate treatment), with 1 point on day 3, 7 points on day 7, and 11 points on day 14 (the reported scores are median values) (*p* < 0.05). Less prominent but comparable results were observed in groups III and V. The worst results, according to the Greenhalgh score, were observed in the group II skin samples, with 1 point on day 3, 4 points on day 7, and 9 points on day 14 (Figure 6).

## 3. Discussion

In this experiment, we looked at the therapeutic potential of *Satureja montana* L. and *Origanum vulgare* L. hydrolates with respect to the skin regeneration process, immune cell infiltration, inflammatory markers, and oxidation levels, for a period of two weeks.

The increasing global burden of antibiotic-resistant bacterial infections, particularly in burn units, poses a critical challenge to modern medicine [1]. Multidrug-resistant organisms (MDROs), such as methicillin-resistant *Staphylococcus aureus* (MRSA), vancomy-cin-resistant *Enterococci* (VRE), and carbapenem-resistant *Enterobacteriaceae* (CRE), frequently colonize burn wounds, leading to severe complications, prolonged hospitalization, and increased mortality [2,3]. This alarming trend necessitates the urgent exploration of alternative or complementary therapeutic strategies. The present study, demonstrating the anti-inflammatory and antioxidant properties of *Satureja montana* L. and *Origanum vulgare* L. hydrolates, is particularly interesting in this context. While our study did not directly assess the antimicrobial activity of the hydrolates against specific bacterial strains, the known broad-spectrum antimicrobial properties of their key constituents, like carvacrol and thymol, against both Gram-positive and Gram-negative bacteria, including antibiotic-resistant strains, suggest a potential indirect benefit in preventing or mitigating burn wound infections [12,13,14].

*Satureja montana* L. (winter savory) and *Origanum vulgare* L. (oregano) are aromatic plants belonging to the *Lamiaceae* family, renowned for their rich content of bioactive compounds and extensive traditional use in folk medicine for treating various ailments, including skin disorders and infections. These plants have been particularly valued in Mediterranean and Balkan regions for their antimicrobial, anti-inflammatory, and wound-healing properties, making them ideal candidates for modern phytotherapeutic applications in burn wound management [15].

The therapeutic application of plant-derived compounds in wound healing is a rapidly expanding field. Numerous studies have investigated the efficacy of various plant ex-tracts, essential oils, and their formulations in treating burns and other skin injuries. For example, research on *Aloe vera* by Maenthaisong et al. [16] demonstrated its wound healing properties, including anti-inflammatory and antimicrobial effects, often attributed to polysaccharides and glycoproteins. Similarly, propolis, a resinous mixture collected by honeybees, has shown promising results in burn wound healing due to its antibacterial, antioxidant, and anti-inflammatory activities [17]. While these studies often utilize different plant species and extraction methods (e.g., alcoholic extracts, ointments), the over-arching principle of leveraging natural compounds for their synergistic therapeutic effects remains consistent with our findings.

For instance, research by Burt [18] highlighted the potent antibacterial effects of essential oils, including those rich in carvacrol and thymol, against a wide range of food-borne pathogens. Similarly, Chouhan et al. [19] reviewed the antimicrobial activity of various essential oils, emphasizing their potential as alternatives to conventional antibiotics. These studies, while focusing on essential oils, provide a strong theoretical basis for the antimicrobial potential of hydrolates, given that they contain water-soluble fractions of these bioactive compounds. The observed reduction in inflammatory infiltration (CD45+ cells) and accelerated wound closure in our study could indirectly contribute to a reduced susceptibility to infection, as a healthy, rapidly healing wound provides a less hospitable environment for bacterial proliferation.

Our use of hydrolates, specifically, offers distinct advantages in wound care. Hydrolates are aqueous by-products of essential oil distillation, containing water-soluble aromatic compounds that are often less concentrated but can be less irritating and more suitable for topical application on sensitive tissues, like burn wounds, compared to concentrated essential oils [20]. Jakubczyk et al. extensively reviewed the antioxidant properties, chemical composition, and potential applications of plant hydrolates, underscoring their therapeutic value [21]. This aligns with our observation that *Satureja montana* L. and *Origanum vulgare* L. hydrolates effectively modulated redox status and inflammation, suggesting that even the water-soluble components possess significant bioactivity.

Gas chromatography–mass spectrometry (GC–MS) analyses of these hydrolates have consistently revealed the presence of key bioactive compounds, albeit in different concentrations compared to their corresponding essential oils. According to Aćimović et al. [22], the volatile fraction of *Satureja montana* L. hydrolates contains carvacrol as the predominant compound (ranging from 41.5% to 69.99%), along with its biosynthetic precursors p-cymene (3.69% to 9.69%) and γ-terpinene (1.51% to 5.92%). Additionally, compounds, such as β-caryophyllene, camphor, and linalool, contribute to the overall therapeutic profile. Our analysis generally confirmed these observations, showing carvacrol as the dominant constituent of *Satureja montana* L. hydrolate (89.2%), with p-cymene (3.1%), thymol (2.8%), and γ-terpinene (1.6%) also present in proportions comparable to data in the literature. From a pharmacognostic perspective, the prevalence of monoterpenoid compounds, such as carvacrol, thymol, and their biosynthetic precursors, is particularly relevant, as these molecules are known to contribute to antioxidant defense, the modulation of inflammatory pathways, and the stimulation of reparative regeneration in damaged skin.

Similarly, research on *Origanum vulgare* L. hydrolates has shown that carvacrol (51.4% to 63.97%) remains the major constituent, followed by p-cymene (6.73% to 12.63%) and linalool (3.67%) [23]. Importantly, hydrolates differ from essential oils in their chemical composition due to the water solubility of certain aromatic compounds. Studies have demonstrated that hydrolates contain higher percentages of oxygenated compounds (98.3–99.8%) compared to essential oils (34.8–69.6%) [21], making them particularly rich in water-soluble phenolic compounds, flavonoids, and other polar molecules that contribute to their antioxidant and anti-inflammatory properties. The GC–MS findings are broadly consistent with these reports, showing carvacrol as the major constituent of *Origanum vulgare* L. hydrolate (43.7%), although at a somewhat lower level compared to published ranges. Interestingly, thymol was detected in a notable proportion (18.6%), suggesting a more balanced distribution of phenolic monoterpenes. Alongside p-cymene (9.4%) and γ-terpinene (7.8%), these compounds, together with minor constituents, such as sabinene, linalool, and α-pinene, collectively shape a phytochemical profile that may underlie the observed antioxidant and anti-inflammatory potential.

Morphological analysis using routine (hematoxylin and eosin) and Masson’s tri-chrome staining revealed the most intensive wound regeneration process in group IV (*Satureja montana* L. hydrolates. The process was less prominent in groups III (conventional treatment) and V (*Origanum vulgare* L. hydrolates); the least favorable results were seen in group II (no treatment).

Immunohistochemical staining with antibodies to CD45/LCA showed extensive immune infiltration of burn injury site in all groups on day 3, with a prominent subsequent decrease in CD45+ cell density in groups III–V. The most favorable results were seen in group IV and V— with considerably lower immune-positive cell density on day 7 and 14.

Using ELISA, we found that all burned animals had much more malondialdehyde (MDA) in their skin. This means that lipid peroxidation and oxidative stress were occurring at a significant rate, in the wound. However, use of the herbal treatments with *Origanum vulgare* L., and especially *Satureja montana* L., lowered the MDA levels considerably. In addition, we found that the burned skin had lower levels of antioxidant enzymes like superoxide dismutase (SOD) and glutathi-one (GSH). Group IV, treated with *Satureja montana* L. hydrolates, had the smallest drop in SOD and GSH compared to the control group. By day 14, the SOD and GSH levels in this group were almost back to normal.

However, we found that *Satureja montana* L. hydrolates modulated levels of pro- and counter-inflammatory cytokines, decreased levels of IL-1 and TNF-α, and a reciprocally upregulated IL-10 level at the burn injury site, realizing its counter-inflammatory effect.

The results show a strong link between the redox status in burn wounds and the type of treatment used. The group treated with *Satureja montana* L. hydrolates (group IV) had redox levels closest to normal, which also matched how quickly the burn wound healed. This was further supported by the integrated wound healing assessment using the Green-halgh score and macroscopic observations, which collectively indicated an accelerated healing process.

While our study provides interesting data for the antioxidant and anti-inflammatory effects of these hydrolates, we did not directly assess their antibacterial activity. The observed improvements in wound healing are likely attributable to the modulation of oxidative stress and inflammation, which are key drivers of tissue damage in burn wounds. However, the known antimicrobial properties of carvacrol and thymol [8,9] may provide an additional, indirect benefit by preventing secondary infections, which are a major complication in burn patients.

The medicinal effects of *Satureja montana* L. and *Origanum vulgare* L. derive from the carvacrol, gamma-terpinene, p-cymene, and thymol they contain. Studies show that the highest concentration of these substances in herbal remedies is achieved through steam distillation, resulting in hydrolates [22,23]. These substances kill bacteria by breaking down bacterial cell membranes; they stop bacteria from growing by suppressing the activity of bacterial ATPases [10]. In addition, these extracts reduce inflammation and act as antioxidants. They fight inflammation by blocking COX-1, COX-2, lipoperoxidase, and met-alloproteinases. They also suppress the pro-inflammatory transcription factor NF-κB, responsible for IL-1 and TNF-α synthesis, thereby preventing excessive tissue damage from inflammation [5,24,25]. However, the most important effect in healing burns is the antioxidant activity of these compounds. Burns cause a huge increase in reactive oxygen species (ROS) in wound tissues, which weakens the local immune system [26]. This occurs because high levels of ROS stimulate Fas-mediated suppression of Akt1 in immune cells, reducing their activity through negative feedback. Akt1 is a key regulator of cell activity. When Akt1 is not active enough, immune cells become less effective at fighting microbes and are more likely to die. The antioxidant activity of carvacrol and thymol breaks this cycle, boosts Akt1 activity, and increases the antimicrobial activity and survival of monocytes and granulocytes. Increased Akt1 expression in epidermal cells increases their proliferation, which speeds up wound re-epithelialization [27].

**Figure 7:**
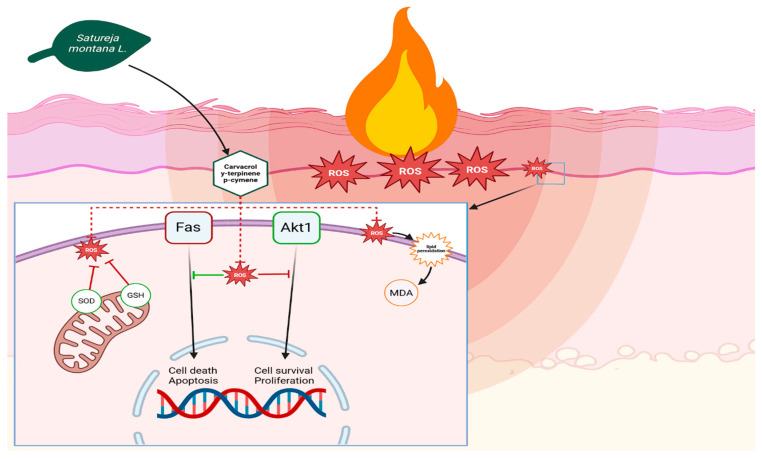
Schematic representation of the proposed effect of *Satureja montana* L. compounds on the redox status of burn wound: SOD—superoxide dismutase; MDA—malondialdehyde; GSH—glutathione. Green arrows represent enhancement, and red arrows represent suppression.

While our study did not directly investigate the molecular mechanisms of Akt1 involvement, the observed improvements in wound healing and inflammatory markers are consistent with the known roles of these compounds in modulating cellular pathways, as supported by existing studies in the literature [26,27]. It is highly probable that the complex biological activities of *Satureja montana* L. and *Origanum vulgare* L. hydrolates involve a broader spectrum of interconnected molecular pathways (Figure 7).

### Rabbit Model Selection

The selection of New Zealand White rabbits as the experimental model for this burn wound healing study was based on several anatomical, physiological, and practical considerations that make this species particularly suitable for translational research in dermatology and wound healing.

From a pathohistological perspective, rabbit skin shares similarities with human skin that are crucial for burn wound research. The dermal thickness of skin in rabbits (1.5–2.0 mm) closely approximates that of human skin (1.5–4.0 mm), particularly in areas commonly affected by burns [28]. This similarity is critical because burn depth classification and healing mechanisms are directly related to dermal thickness and structure. Further-more, the ratio of dermis to epidermis in rabbits (approximately 10:1) is comparable to humans (approximately 8–12:1), ensuring that burn injuries penetrate similar proportion-al depths of skin layers [29].

The vascular architecture of rabbit skin also closely resembles that of humans. Both species possess a similar dermal vascular plexus organization, with superficial and deep plexuses connected by vertical vessels [30]. This vascular similarity is particularly important for burn wound studies because thermal injury primarily affects the microvascular network; the healing response is heavily dependent on angiogenesis and revascularization patterns.

Rabbit dermal collagen consists primarily of type I collagen (approximately 80–85%) with type III collagen (10–15%), which closely matches the human dermal collagen profile [31]. This similarity is essential for burn wound healing studies because collagen remodeling is a central component of the healing process; the response to thermal injury and subsequent treatment interventions depends heavily on the baseline collagen architecture.

This study should be viewed as a pilot proof-of-concept project, designed to test the hypothesis that hydrolates of *Satureja montana* L. and *Origanum vulgare* L. exert anti-inflammatory and antioxidant effects in burn wound healing. The present results support our hypothesis and indicate that hydrolates can beneficially modulate oxidative stress, cytokine balance, and local immune cell infiltration. Nevertheless, to fully elucidate their therapeutic potential, further research is essential.

Future directions should include molecular and genetic studies to clarify the cellular pathways involved, as well as in vitro experiments on skin cell cultures and ex vivo investigations on human tissue samples. Expanding preclinical studies to additional animal models, particularly rodents and porcine skin (the gold standard in translational dermatology), will be critical for validating the reproducibility and scalability of the observed effects. Importantly, progress in this field requires multidisciplinary collaboration, involving dermatologists, pharmacologists, and other specialists. We think that the following integration will provide both mechanistic insights and a practical basis for translating phytotherapeutic hydrolates into future clinical trials aimed at improving burn care.

## 4. Material and Methods

### 4.1. Experimental Animals

Male rabbits (3.0 kg ± 330 g; age: 7–8 weeks) were housed in a standard controlled vivarium environment, maintained at a stable temperature of 22–23 °C with a 12 h light/dark cycle (12L:12D). The humidity was regulated between 40–60%; the rabbits had free access to standard laboratory chow and water ad libitum. The animals were housed in pairs within stainless steel cages, lined with absorbent material (rice husk) to provide suitable nesting materials and to reduce the stress associated with solitary confinement, which could influence their behavior and physiological responses.

### 4.2. Experimental Design

In this experiment, animals were divided into five groups based on the type of intervention: Group I—control (n = 5), animals without skin burn injury and without treatment; Group II—burn wound model (n = 5), animals with a burn wound but without treatment; Group III—conventional treatment model (representative of practices in some CIS countries) (n = 5), animals with a burn wound, treatment was performed with Levomekol liniment (methyluracil, chloramphenicol), boric acid, and gauze dressings soaked in Betadine (povidone-iodine); Group IV—burn wound phytotherapy model (n = 5), treatment was performed with *Satureja montana* L. hydrolate; and Group V—burn wound phytotherapy model (n = 5), treatment was performed with *Origanum vulgare* L. hydrolate. Baseline assessments encompassed weight measurements, behavioral evaluations, and biochemical analyses to exclude animals exhibiting physiological deviations.

**Figure 8:**
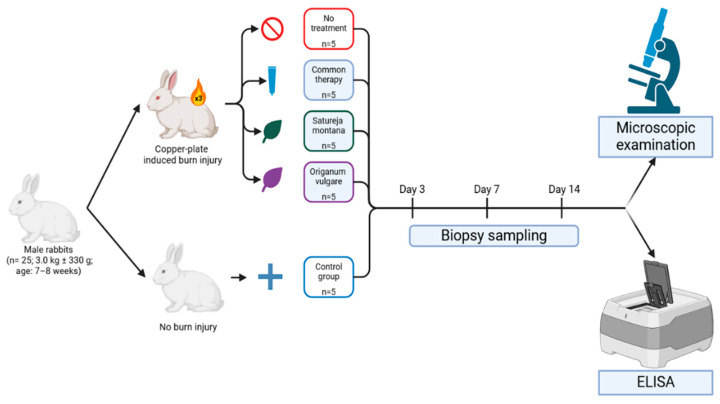
Schematic representation of experimental design.

Burn injury site samples were taken at day 3, 7, and 14 post injury (Figure 8). Excision was carried out using surgical forceps, scalpel, and fine-tip scissors, followed by wound cleaning with a 0.5% chlorhexidine solution (within healthy tissue margins only; burn tissue was not manipulated, and disinfection was carried out after excision). In cases of unresolvable complications, animals were euthanized using Zoletil^®^ 100 (Tiletamine and Zolazepam; Virbac AH, Inc., Fort Worth, TX, USA) (15 mg/kg body weight) and xylazine (5 mg/kg body weight; Nita-Pharm, Saratov, Russia), adhering to all institutional ethical standards for animal care.

The time points for sample collection (days 3, 7, and 14) were chosen to assess the dynamic changes across key phases of burn wound healing: day 3 represents the acute inflammatory phase; day 7 represents the proliferative phase and onset of re-epithelialization; and day 14 represents the remodeling phase and completion of re-epithelialization.

### 4.3. Burn Injury Induction

The thermal burn injury model used in this study involved the induction of a standardized III degree burn on New Zealand White rabbits, specifically sedated with neuroleptanalgesia (Zoletil^®^ 100, Virbac AH, Inc., Fort Worth, TX, USA; administered at a dose of 5 mg/100 g of body weight). The burn wounds were generated by applying a pre-heated copper plate (2 × 3 cm^2^, 200 g) brought to a red-hot state over an alcohol flame. The plate was carefully positioned on the pre-shaved dorsal surface of the rabbits with an applied standardized pressure for 30 s. To enable dynamic monitoring of the healing process within each individual, four identical burns were sequentially created along the animal’s back, extending from the interscapular region toward the tail.

### 4.4. Hydrolate Extraction

Hydrolates of *Satureja montana* L. and *Origanum vulgare* L. were obtained by steam distillation using a laboratory-scale Clevenger-type apparatus (Duran Group GmbH, Mainz, Germany). Fresh aerial parts of each plant (500 g) were subjected to distillation with deionized water (1:5 *w*/*v* ratio) for 3 h at atmospheric pressure (100 °C) using a heating mantle (IKA-Werke GmbH & Co. KG, Staufen, Germany). The vaporized constituents were condensed using a Liebig condenser (Schott AG, Mainz, Germany) and collected as a biphasic distillate, from which the aqueous phase (hydrolate) was separated using a separatory funnel (Pyrex, Corning Inc., New York, NY, USA) and used for further processing.

### 4.5. Product Safety

To guarantee batch-to-batch consistency and product safety, a multi-level validation protocol was implemented. Plant species were taxonomically identified and authenticated by a certified botanist, with voucher specimens deposited in the herbarium of the Institute of Translational Medicine and Biotechnology, Sechenov University, Moscow, Russia. Distillation parameters including time, temperature, pressure, and plant-to-water ratio were rigorously controlled for reproducibility using a digital thermometer (Testo SE & Co. KGaA, Lenzkirch, Germany) and pressure gauge (WIKA Alexander Wiegand SE & Co. KG, Klingenberg, Germany). Each hydrolate batch was evaluated for clarity through visual inspection; pH measurement using a digital pH meter (Hanna Instruments, Woonsocket, RI, USA) with a range of 3.2–6.0; density determination using a digital densi-tometer (Anton Paar GmbH, Graz, Austria); and refractive index measurement with an Abbe refractometer (ATAGO Co., Ltd., Tokyo, Japan).

### 4.6. Microbiological and Chemical Purity

The microbiological purity assessment was conducted according to European Pharmacopoeia standards using sterile sampling techniques and incubation in a microbiological incubator (Thermo Fisher Scientific, Waltham, MA, USA) for total bacterial count, molds, and yeasts determination. Only sterile, contamination-free hydrolates meeting pharmacopeial specifications were used for the subsequent gel preparation. Chemical profiling was performed using gas chromatography–mass spectrometry (GC-MS) on an Agilent 7890A GC system coupled with a 5975C MSD (Agilent Technologies, Santa Clara, CA, USA) to identify and quantify major constituents, confirming the expected composition and validating the extraction efficacy [32].

### 4.7. Dosage Form Preparation

For the therapeutic gel preparation, 2% hydroxyethyl cellulose (HEC; Sigma-Aldrich, St. Louis, MO, USA) at a concentration of 20 g/L was gradually dispersed in the validated hydrolates under continuous stirring using a magnetic stirrer (IKA-Werke GmbH & Co. KG, Staufen, Germany) at room temperature. The mixture was then gently heated to 40 °C using a water bath (Julabo GmbH, Seelbach, Germany) to achieve full hydration and homogeneity while monitoring the temperature with a digital thermometer (Testo SE & Co. KGaA, Lenzkirch, Germany). The resulting gels were transparent to slightly opalescent and transferred to sterile, airtight containers using sterile techniques in a laminar flow hood (Thermo Fisher Scientific, Waltham, MA, USA). Final gel products were stored at 4 °C in a pharmaceutical refrigerator (Liebherr Group, Bulle, Switzerland) to preserve stability, microbial purity, and bioactivity throughout the experimental period.

### 4.8. Assessment of Oxidative Stress and Inflammatory Markers

The skin tissue sample homogenate was prepared by homogenizing 1 g of tissue in 4.5 mL of cold 0.1 M potassium buffer (pH 7.4). The mixture was then centrifuged at 13,000 rpm for 10 min at 4 °C. The resulting supernatant was stored at −80 °C for further analysis. The following levels were evaluated using enzyme-linked immunosorbent assay (ELISA) kits (Lifespan Biosciences, Seattle, WA, USA), according to the manufacturer’s protocols: malondialdehyde (MDA), a biomarker of lipid peroxidation; superoxide dismutase (SOD), an antioxidant enzyme; glutathione (GSH), a key mediator of the glutathione antioxidant system; interleukin-1 (Il-1) and tumor necrosis factor alpha (TNF-α), main pro-inflammatory markers; and interleukin-10 (Il-10), a counter-inflammatory cytokine in the skin homogenate. The MDA assay employed a competitive ELISA format recognizing MDA–protein adducts. SOD, GSH, Il-1, TNF-α and Il-10 were quantified by antigen-specific immunoassays calibrated to kit standards. All ELISAs were read at 450 nm with a 4-parameter logistic fit; samples were assayed in duplicate.

The sensitivities (detection limits) of the ELISA kits were as follows: IL-1β (3.6 pg/mL), TNF-α (4.2 pg/mL), and IL-10 (9.4 pg/mL), as specified by the manufacturer. To express cytokine levels as ng/g of tissue, the measured concentrations (pg/mL) were converted using the following formula:

$$\frac{\left(cytokine\;concentration\;in\;pg/mL\;of\;homogenate\;\times \;homogenization\;volume\;in\;mL\right)}{(tissue\;weight\;in\;g)/1000}.$$

### 4.9. Morphological Study

After the collection of biopsy samples at specified time points, the excised skin samples were assessed macroscopically. The severity of the burn was assessed as grade III if it was red or brown in color and had a dry, inelastic, leathery surface, after which the material was sent for enzyme immunoassay. Biopsy samples from burn wounds were taken at all the aforementioned time points. Histological slides were made with a common laboratory technique: preservation in 10% formalin solution (neutral-buffered, pH 6.8–7.2), subsequent dehydration using a graded series of alcohols, and paraffin embedding. For the histological examination, thin sections (3 μm) were prepared and stained using hematoxylin and eosin (H and E) and Masson trichrome staining (Weigert’s hematoxylin, acid fuchsin, phosphomolybdic acid, and aniline blue). The assessment of the degree of fibrosis was carried out in points, taking into account the area and optical density (staining of fibers according to Masson) in relative units: “0”—absent; “1”—weak (0–0.3; <25%); “2”—moderate (0.3–0.6; 25–50%); “3”—strong (0.6–0.9; 50–75%); and “4”—pronounced (>0.9; >75%). Integrative wound healing evaluation was carried out using the Greenhalgh score at the aforementioned control points.

The Greenhalgh score, as described by Greenhalgh et al. [33], is a composite scoring system used to assess burn wound healing. It evaluates three parameters: (i) wound contraction; (ii) re-epithelialization; and (iii) granulation tissue formation. Each parameter is rated on a scale from 0 (absent) to 4 (complete/optimal); the scores are summed to yield a total score ranging from 0 to 12, with higher scores indicating more advanced healing.

### 4.10. Immunohistochemical Examination

Immunohistochemical staining was performed according to a standard protocol on a Ventana Benchmark XT (universal staining system; Ventana NexES^®^ software v5.0) using the ultraView Universal DAB Detection Kit (Ventana Medical Systems, a member of the Roche Group, Tucson, AZ, USA) with antibodies against CD34/LCA (clone 2B11 + PD7/26 Cell Marque) for skin samples from groups II–V. Human lymph node fragments were used as an external control. Slides were counterstained with hematoxylin.

Quantification of CD45+ cells was performed in QuPath (v0.3.2). Multiple non-overlapping fields were analyzed to cover a total area of 1 mm^2^ per sample; positive cells were detected by DAB thresholding with standardized parameters. Results were reported as CD45+ cells per mm^2^.

### 4.11. Statistical Analysis

Statistical analysis of the sample was performed using STATISTICA 13.5.0.17 software (TIBCO Software Inc., Palo Alto, CA, USA). The Shapiro–Wilk test was used to assess the normality of the data distribution. For comparisons between study groups with non-normal distributions, the Kruskal–Wallis test followed by Dunn’s post hoc test was applied. Effect sizes (epsilon-squared for Kruskal–Wallis and rank-biserial correlation for Mann–Whitney U test) and, where appropriate, bootstrapped confidence intervals or interquartile ranges, were calculated to provide a comprehensive understanding of the magnitude and precision of observed effects. Multiple comparisons were performed using the Mann–Whitney U test. A *p*-value ≤ 0.05 was considered statistically significant.

## 5. Conclusions

In our study, we found that the use of hydrolates based on *Origanum vulgare* L. and, especially, *Satureja montana* L. in skin biopsy samples with a burn injury, have pronounced counter-inflammatory effect, antioxidant activity and pro-regenerative properties compared to conventional burn injury treatment.

Treatment with hydrolates reduced lipid peroxidation (MDA) while restoring antioxidant defenses (SOD and GSH). Pro-inflammatory cytokines (IL-1β, TNF-α) declined, whereas the counter-inflammatory cytokine (IL-10) increased, most prominently with *Satureja montana* L. Immune infiltration (CD45+ cells) decreased in parallel with improved histological findings, including re-epithelialization, reduced inflammation, and collagen reorganization.

To translate these preclinical findings into clinical practice, future research should focus on detailed mechanistic investigations and well-designed clinical trials to establish safety, efficacy, and applicability in human patients.

## Figures and Tables

**Table 1:** Chemical composition of *Satureja montana* L. hydrolate.

Compound	Retention Index (RI)	Concentration (%)
Carvacrol	1309	89.2
Thymol	1287	2.8
Linalool	1092	1.3
Borneol	1157	1.2
Terpinen-4-ol	1171	0.9
p-Cymene	1019	3.1
γ-Terpinene	1050	1.6
1-Octen-3-ol	972	0.7
Myrcene	983	0.8
α-Terpineol	1185	0.6
1,8-Cineole	1030	0.5
Camphor	1146	tr

Notes: RI values were calculated relative to n-alkanes; concentrations are expressed as relative peak area percentages; tr—indicates trace amounts (<0.1%).

**Table 2:** Chemical composition of *Origanum vulgare* L. hydrolate.

Compound	Retention Index (RI)	Concentration (%)
Carvacrol	1298	43.7
Thymol	1292	18.6
p-Cymene	1025	9.4
γ-Terpinene	1058	7.8
Sabinene	969	2.7
Linalool	1093	2.1
β-Myrcene	988	1.6
α-Pinene	934	0.9
Terpinen-4-ol	1174	1.1
α-Terpinolene	1051	0.6
1,8-Cineole	986	0.4
Camphor	952	tr

Notes: RI values were calculated relative to n-alkanes; concentrations are expressed as relative peak area percentages; tr—indicates trace amounts (<0.1%).

## Data Availability

Data is contained within the article.

## References

[1] Jeschke M.G., Van Baar M.E., Choudhry M.A., Chung K.K., Gibran N.S., Logsetty S. (2020). Burn injury. Nat. Rev. Dis. Primers.

[2] Tredget E.E., Shankowsky H.A., Rennie R., Burrell R.E., Logsetty S. (2004). Pseudomonas infections in the thermally injured patient. Burns.

[3] Keen E.F., Robinson B.J., Hospenthal D.R., Aldous W.K., Wolf S.E., Chung K.K., Murray C.K. (2010). Prevalence of multidrug-resistant organisms recovered at a military burn center. Burns.

[4] Dogra A., Kotwal P., Gour A., Bhatt S., Singh G., Mukherjee D., Nandi U. (2020). Description of Druglike Properties of Safranal and Its Chemistry behind Low Oral Exposure. ACS Omega.

[5] Abdelshafeek K.A., Osman A.F., Mouneir S.M., Elhenawy A.A., Abdallah W.E. (2023). Phytochemical profile, comparative evaluation of *Satureja montana* alcoholic extract for antioxidants, anti-inflammatory and molecular docking studies. BMC Complement. Med. Ther..

[6] Cagnoli G., Bertelloni F., Ebani V.V. (2024). In Vitro Antibacterial Activity of Essential Oils from *Origanum vulgare*, *Satureja montana*, *Thymus vulgaris*, and Their Blend Against Necrotoxigenic (NTEC), Enteropathogenic (EPEC), and Shiga-Toxin Producing *Escherichia coli* (STEC) Isolates. Pathogens.

[7] Pino-Otín M.R., Gan C., Terrado E., Sanz M.A., Ballestero D., Langa E. (2022). Antibiotic properties of *Satureja montana* L. hydrolate in bacteria and fungus of clinical interest and its impact in non-target environmental microorganisms. Sci. Rep..

[8] Tullio V., Roana J., Cavallo L., Mandras N. (2023). Immune Defences: A View from the Side of the Essential Oils. Molecules.

[9] Chouhan S., Sharma K., Guleria S. (2017). Antimicrobial Activity of Some Essential Oils—Present Status and Future Perspectives. Medicines.

[10] Abbad I., Soulaimani B., Iriti M., Barakate M. (2025). Chemical Composition and Synergistic Antimicrobial Effects of Essential Oils From Four Commonly Used *Satureja* Species in Combination with Two Conventional Antibiotics. Chem. Biodivers..

[11] Morais A., Lima L., Silva A., Lienou L., Ferreira A., Watanabe Y., Joaquim D., Alves B., Pereira A., Alves D. (2023). Effect of carvacrol antioxidant capacity on oocyte maturation and embryo production in cattle. Zygote.

[12] Al-Tawalbeh D., Alkhawaldeh Y., Sawan H.M., Al-Mamoori F., Al-Samydai A., Mayyas A. (2024). Assessment of carvacrol-antibiotic combinations’ antimicrobial activity against methicillin-resistant *Staphylococcus aureus*. Front. Microbiol..

[13] Angane M., Swift S., Huang K., Perera J., Chen X., Butts C.A., Quek S.Y. (2023). Synergistic antimicrobial interaction of plant essential oils and extracts against foodborne pathogens. Food Sci. Nutr..

[14] Kimura A.H., Dahmer D., Isawa L.A., da Silva A.B.O., Souza L.M.d.S., Takata P.H., Scandorieiro S., Deonas A.N., Germiniani-Cardozo J., Vespero E.C. (2025). Hydrogel Containing Biogenic Silver Nanoparticles and *Origanum vulgare* Essential Oil for Burn Wounds: Antimicrobial Efficacy Using Ex Vivo and In Vivo Methods Against Multidrug-Resistant Microorganisms. Pharmaceutics.

[15] Dimitrijević M., Stojanović-Radić Z., Radulović N., Nešić M. (2025). Chemical Composition and Antifungal Effect of the Essential Oils of *Thymus vulgaris* L., *Origanum vulgare* L., and *Satureja montana* L. Against Clinical Isolates of *Candida* spp.. Chem. Biodivers..

[16] Maenthaisong R., Chaiyakunapruk N., Niruntraporn S., Kongkaew C. (2007). The efficacy of aloe vera used for burn wound healing: A systematic review. Burns.

[17] Manginstar C.O., Tallei T.E., Niode N.J., Salaki C.L., Hessel S.S. (2023). Therapeutic potential of propolis in alleviating inflammatory response and promoting wound healing in skin burn. Phytother. Res..

[18] Burt S. (2004). Essential oils: Their antibacterial properties and potential applications in foods—A review. Int. J. Food Microbiol..

[19] Hyldgaard M., Mygind T., Meyer R.L. (2012). Essential oils in food preservation: Mode of action, synergies, and interactions with food matrix components. Front. Microbiol..

[20] Almeida H.H.S., Fernandes I.P., Amaral J.S., Rodrigues A.E., Barreiro M.-F. (2024). Unlocking the Potential of Hydrosols: Transforming Essential Oil Byproducts into Valuable Resources. Molecules.

[21] Jakubczyk K., Tuchowska A., Janda-Milczarek K. (2021). Plant hydrolates—Antioxidant properties, chemical composition and potential applications. Biomed. Pharmacother..

[22] Aćimović M., Šovljanski O., Pezo L., Travičić V., Tomić A., Zheljazkov V.D., Ćetković G., Švarc-Gajić J., Brezo-Borjan T., Sofrenić I. (2022). Variability in Biological Activities of *Satureja montana* Subsp. *montana* and Subsp. *variegata* Based on Different Extraction Methods. Antibiotics.

[23] Baser K.H.C. (2008). Biological and Pharmacological Activities of Carvacrol and Carvacrol Bearing Essential Oils. Curr. Pharm. Des..

[24] Butnariu M., Quispe C., Herrera-Bravo J., Helon P., Kukula-Koch W., López V., Les F., Vergara C.V., Alarcón-Zapata P., Alarcón-Zapata B. (2022). The effects of thymoquinone on pancreatic cancer: Evidence from preclinical studies. Biomed. Pharmacother..

[25] El Tawab A.M.A., Shahin N.N., Abdelmohsen M.M. (2014). Protective effect of Satureja montana extract on cyclophosphamide-induced testicular injury in rats. Chem. Biol. Interact..

[26] Dryden M. (2018). Reactive oxygen species: A novel antimicrobial. Int. J. Antimicrob. Agents.

[27] Vannella K.M., Wynn T.A. (2017). Mechanisms of Organ Injury and Repair by Macrophages. Annu. Rev. Physiol..

[28] Montagna W., Yun J.S. (1964). The Skin of the Domestic Pig. J. Investig. Dermatol..

[29] Meyer W., Schwarz R., Neurand K. (1978). The Skin of Domestic Mammals as a Model for the Human Skin, with Special Reference to the Domestic Pig. Skin-Drug Application and Evaluation of Environmental Hazards.

[30] Vardaxis N.J., Brans T.A., Boon M.E., Kreis R.W., Marres L.M. (1997). Confocal laser scanning microscopy of porcine skin: Implications for human wound healing studies. Am. J. Anat..

[31] Bailey A.J., Robins S.P., Balian G. (1974). Biological significance of the intermolecular crosslinks of collagen. Nature.

[32] Santos J.D., Coelho E., Silva R., Passos C.P., Teixeira P., Henriques I., Coimbra M.A. (2019). Chemical composition and antimicrobial activity of Satureja montana byproducts essential oils. Ind. Crops Prod..

[33] Greenhalgh D., Sprugel K., Murray M., Ross R. (1990). PDGF and FGF Stimulate Wound-Healing in the Genetically Diabetic Mouse. Am. J. Pathol..

